# Identifying the Co-Curing Effect of an Accelerated-Sulfur/Bismaleimide Combination on Natural Rubber/Halogenated Rubber Blends Using a Rubber Process Analyzer

**DOI:** 10.3390/polym13244329

**Published:** 2021-12-10

**Authors:** Marek Pöschl, Shibulal Gopi Sathi, Radek Stoček

**Affiliations:** Centre of Polymer Systems, Tomas Bata University in Zlín, Třida Tomáše Bati 5678, 760 01 Zlín, Czech Republic; poschl@utb.cz (M.P.); stocek@utb.cz (R.S.)

**Keywords:** rubber, curing, strain sweep, rheometer, rubber process analyzer

## Abstract

The rheometer curing curves of 50/50 blends of natural rubber (NR) and two different halogenated rubbers with a combination of conventional accelerated sulfur (CV) and 3 phr of a bismaleimide (MF_3_) at 170 °C indicates that a co-curing reaction has been taken place between NR and the halogenated rubbers via Diels–Alder reaction. To further confirm whether the co-curing reaction has taken place in the early stage of curing, a complex test methodology was applied with the help of a rubber process analyzer. In this test, the blends with CV and with CVMF_3_ were subjected to cure at 170 °C for a predetermined time so that both the CV and CVMF_3_ cured blends will have the same magnitude of curing torque. It is then cooled down to 40 °C and the storage modulus (G′) was evaluated as a function of strain from 0.5% to 100% at a constant frequency of 1 Hz. The results reveal that the blends cured with CVMF_3_ exhibit a higher G′ due to the enhanced network strength because of the formation of bismaleimide crosslinks than the same cured with only the CV system. The swelling resistance and the mechanical properties of the blends cured with CVMF_3_ were significantly higher than those cured with only the CV system.

## 1. Introduction

Manufactures of rubber products and suppliers of polymers and raw materials are forced to apply predictive and advanced laboratory test methods and experiments when seeking for high-performance elastomers for future rubber products, as well as for a better, overall, understanding of the properties of the materials. Moreover, they are strictly following the environmental requirements for reducing energy consumption for production while keeping constant or increasing the performance of rubber products. The curing of rubber compounds is one of the most important processes and it is almost the final step of the rubber product development technology. Moreover, the process of curing and curing systems will significantly influence the final performance of rubber products as well as the total time required for rubber production. Through curing, the entangled rubber chains turn into a network structure due to the formation of chemical crosslinks between the rubber chains.

Scientists and technologists have mechanically connected the progressive enhancement of the stiffness of the rubber stocks during curing and developed cure meters to precisely monitor the curing process. In the rubber industry, cure meters are commonly called rheometers. Oscillating disc rheometer (ODR), oscillating die (moving die) rheometer (MDR) and rubber process analyzer (RPA) are the commonly used equipment to characterize the curing behavior of compounded rubber stocks. This equipment can directly describe the kinetics of the crosslinking reaction due to the combination of mechanical representations of chemical processes. The ability of these cure meters to detect the minor changes in different batches of rubber compounds due to improper mixing, insufficient quantity of the compounding ingredients makes it a widely accepted production control instrument in the rubber industry [1,2]. Nowadays, the rheometers are modified in such a way that they can be utilized to test the curing behavior as well as the viscoelastic properties on the same sample to be tested. RPA is one such version of a modified rheometer that can be used to test the curing behavior as well as the viscoelastic properties of the sample after curing. It uses a rotorless biconical die design. The lower die of RPA can oscillate from 0.05°of arc to 90° of arc at an oscillation frequency of 0.1 to 2000 cycles per minute (0.33 Hz to 33 Hz). The temperature can be programmed to change upward or downward between 40 °C and 230 °C. Because of the possibilities of applying a wide range of strains and frequencies to the test sample, RPA can be employed to evaluate the viscoelastic properties of the rubber compounds after the curing has been completed [2,3,4]

It is well-known that natural rubber (NR) is a non-polar, highly unsaturated elastomer. Because of its non-polar nature, NR exhibits poor resistance to hydrocarbon solvents, oils and greases [5]. On contact with these substances, the NR-based compounds undergo failure due to swelling. Therefore, NR is not considered a material of choice for the development of oil seals or gaskets. Moreover, the unsaturated chemical structure of NR makes it vulnerable to weather elements such as oxidative ageing and ozone attack [6]. To overcome the above-mentioned limitations, NR is frequently blended with polar elastomers such as chloroprene rubber (CR) [7,8,9,10] or relatively less polar and less unsaturated elastomers such as bromobutyl rubber (BIIR), etc., [11] and curing the same with proper curing agent. It is well-known that the most appropriate curing system for the NR is the accelerated-sulfur system. The accelerated sulfur system is a package which comprises sulfur, accelerator, activator (Znic oxide, ZnO) and a fatty acid (stearic acid). The accelerated sulfur system produces sulfidic crosslinks in the cured network. Generally, three types of sulfidic crosslinks such as monosulfidic (C–S–C), disulfidic (C–S–S–C), and polysulfidic (C–Sx–C) have been identified in the cured network of NR after curing with the accelerator sulfur system. By adjusting the accelerator to sulfur (A/S) ratio, the level of mono, di, and the polysulfidic crosslinks in the cured network can be manipulated. The conventional accelerated-sulfur vulcanization (CV) system generally produces a cured network with 95% poly and di sulfidic crosslinks and 5% monosulfidic. Therefore, for a CV system, the accelerator to sulfur ratio should maintain in the range 0.1–0.6. This means in the CV system, the sulfur dose is around 2–3.5 phr and the accelerator dose is around 0.4–1.2 phr. [12,13,14]. Unlike NR, both CR and BIIR are generally cured with ZnO. However, some amount of magnesium oxide (MgO) is also used along with ZnO to cure CR. The cured network of CR or BIIR mainly consists of C–C crosslinks [15,16,17]. It is very important to note that being a polar elastomer, CR has many unique properties such as oil, ozone and weather resistance. Similarly, the bromobutyl rubber also possesses resistance to ageing and weathering from atmospheric exposure due to its predominantly saturated polyisobutylene backbone of the butyl rubber. Moreover, bromobutyl rubber possesses low gas and moisture permeability. Therefore, blending of NR with CR or BIIR can exploit certain unique properties of these individual elastomers. However, because of the microstructural differences and the cure rate incompatibility, the blending and curing of NR with either CR or BIIR is very difficult. Therefore, a chemical that can act as a reactive compatibilizing or co-curing agent between NR and CR (or BIIR) is essential for enhancing the compatibility between NR and CR or BIIR.

In our previous article, the co-curing effect of a combination of conventional accelerated sulfur (CV) and a bismaleimid on a 50/50 blend of NR/CR was reported based on a moving die rheometer (MDR-3000, Mon Tech, Buchen, Germany). Mainly based on this rheometer cure data, a mechanism responsible for the co-curing effect of the CV/bismalimide on the NR/CR blend has been proposed [18]. In the present work, the curing behavior of a 50/50 blend of NR with another grade of CR and a 50/50 blend of NR with another halogenated rubber was investigated; BIIR was again investigated in the presence of a combination of CV/bismaleimide using the same MDR. For a comparative analysis, the curing behavior of neat NR with CV system and the curing behaviors of neat CR and BIIR with metal oxides were also reported. To check the validity of the curing behavior and the mechanism proposed in reference [18], a specially designed testing protocol was employed in this study with the help of a rubber process analyzer (RPA).

## 2. Materials

**Figure 1:**
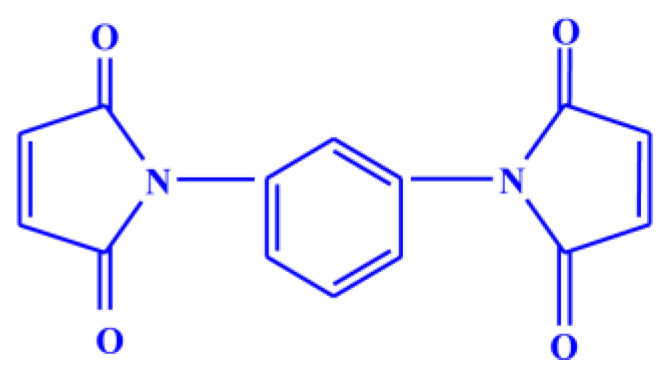
Chemical structure of *N*,*N*′-meta phenylene dimaleimide (Maleide F).

The natural rubber (standard Vietnamese rubber with a Mooney viscosity ML (1 + 4 at 100 °C: 60 ± 5) was obtained from Binh Phuoc, Vietnam under the trade name SVR CV60, Chloropre rubber (Chloroprene Denka M.40), DuPont elastomer with a Mooney viscosity ML (1 + 4, 100 °C: 48 ± 5) and Bromobutyl rubber (Exxon bromobutyl 224) with a Mooney viscosity ML (1 + 8, 125 °C: 46 ± 5) were used as the base elastomers. Maleide F (MF) is a combination of 75% *N*,*N*′-meta phenylene dimaleimide and a 25% blending agent was procured from Krata Pigment, Tambov, Mentazhnikov, Russia. The chemical structure of MF is shown in Figure 1. Other ingredients such as sulfur; n-cyclohexyl-2-benzothiazole sulfenamide (CBS); stearic acid; and zinc oxide; and magnesium oxide were purchased from Sigma-Aldrich, Prague, Czech Republic.

### 2.1. Preparation of Rubber Compounds

The formulations of the mixes with designations are displayed in Table 1. All the compounds were prepared using an internal mixer (Brabender Plastograph, GmbH & Co. KG, Duisburg, Germany) with a chamber volume of 50 cc. A fill-factor of 0.8 was taken for the efficient mixing of the ingredients. To prepare the NR-based compound, the neat NR was masticated at 50 °C under 50 rpm for 2 min. To this, the ZnO, stearic acid, and MF were added, and the mixing was continued under the same rotor speed and temperature for another 2 min. The sulfur and CBS were then added and mixed for additional one minute. After the mixing, the compound was discharged and homogenized using a two-roll mill. Similarly, the CR- and the BIIR-based compounds were prepared after masticating them at 50 °C under 50 rpm for 2 min. To the masticated CR, the ZnO, MgO, stearic acid and MF were added, and the mixing was continued for another 2 min. To the masticated BIIR, only the ZnO and MF were added and mixed for 2 more minutes. Later, the mixes were discharged and homogenized using two-roll mill as in the case of NR. To prepare the blend-based compounds, the individual rubbers were masticated separately for 2 min under the same processing conditions. The pre-masticated rubbers were mixed for 1 min. To this, the ZnO, MgO, stearic acid and MF were added, and the mixing was continued for 2 more minutes. Finally, the sulfur and CBS were added and mixed for an additional minute. After the mixing, the compound was discharged and homogenized using a two-roll mill. It was then molded into sheets with a thickness of 2 mm by applying a constant force of 200 N using a compression molding heat press LaBEcon 300 (Fontijne Presses, Delft, The Netherlands). To avoid the interference of reversion, a molding temperature of 170 °C was selected in this study.

### 2.2. Characterization

#### 2.2.1. Cure Characteristics

Maximum torque: *M_H_,* minimum torque: *M_L_,* the difference between maximum and minimum torque: Δ*M*, scorch time: *T*_*S*2_, optimum cure time: *T*_90_ (the time required for the torque to reach 90% of the maximum torque) of the rubber compounds were determined from the cure curves from a moving die rheometer (MDR-3000, Mon Tech, Buchen, Germany) at 170 °C as per ASTM D 5289. The cure rate index (CRI), a measure of the rate of curing, was calculated using Equation (1).

CRI = 100/(*T*_90_ − *T*_*S*2_) (1)

#### 2.2.2. Cure-Strain Sweep Analysis

Being a viscoelastic material, rubber possess both the elastic (G′) and the viscous (G″) moduli. After curing, the elastic component will be the predominant one. Therefore, G′ can give an idea of the strength of the cured network. Generally, the higher the G′, the higher will be the strength of the cured network. Using a rubber process analyzer (RPA) it is possible to measure both G′ and G″ of rubber compounds at a wide range of temperatures, strains and frequencies. To compare the strength of the blends cured with CV and CV/bismaleimide, a special test configuration in the form of ‘cure-strain sweep’ was created using a Premier RPA (Alfa Technologies, Hudson, OH, USA). In this test, the uncured sample was subjected to cure up to a predetermined time. After this, the sample is cooled down to 40 °C within the cavity of RPA die and we conducted a strain sweep experiment by varying the strain from 0.5% to 100% at a constant frequency of 1 Hz.

#### 2.2.3. Swelling Behavior

Samples with a dimension of 20 mm × 30 mm × 2 mm size with an initial weight (*W_i_*) were swelled in toluene at room temperature until they reached an equilibrium state of swelling. The swelled samples were then taken out and the adhered solvent was wiped off from the surface using a filter paper, and the weights (*W_s_*) were immediately recorded. From the values of *W_i_* and *W_s_*, and the molecular weight of the solvent (*M_w_*), the equilibrium swelling in percentage and the solvent uptake in mol percentage were calculated using Equations (2) and (3), respectively, [9,19]. To understand the speed of swelling, the solvent uptake of the blends cured with CV and CV/bismaleimide were also measured at different time intervals from 0 to 2880 min.

(2) $$Equilibrium\;swelling\;(\%)=\frac{Ws-Wi}{Ws}\times 100$$

(3) $$\mathrm{Solvent}\;\mathrm{uptake}\;(\mathrm{mol}\%)=\frac{1}{Mw}\left(\frac{Ws-Wi}{Wi}\right)\times 100\;$$

#### 2.2.4. Mechanical (Tensile) Properties

The stress-strain behavior and the corresponding tensile properties of the vulcanizates of the blends were measured using a universal testing machine (Testometric M350, Testometric Company, Ltd., Rochdale, UK). The testing was performed under ambient conditions at a crosshead speed of 500 mm/min as per ISO 37 using S2 type specimen with a thickness of 2 mm. The results were reported at an average of six tested specimens. 

#### 2.2.5. Hardness Testing

Cured samples having smooth surfaces were used to measure the indentation hardness using a Shore-A hardness tester (Bareiss Durometer, Oberdischingen, Germany) as per ASTM D 2240. Indentations were made on different areas of the samples by applying constant pressure for 3 s. Five readings were taken from different areas of the sample, and we reported the average value. 

## 3. Results and Discussion 

### 3.1. Curing Behavior of Neat NR with CV and MF

**Figure 2:**
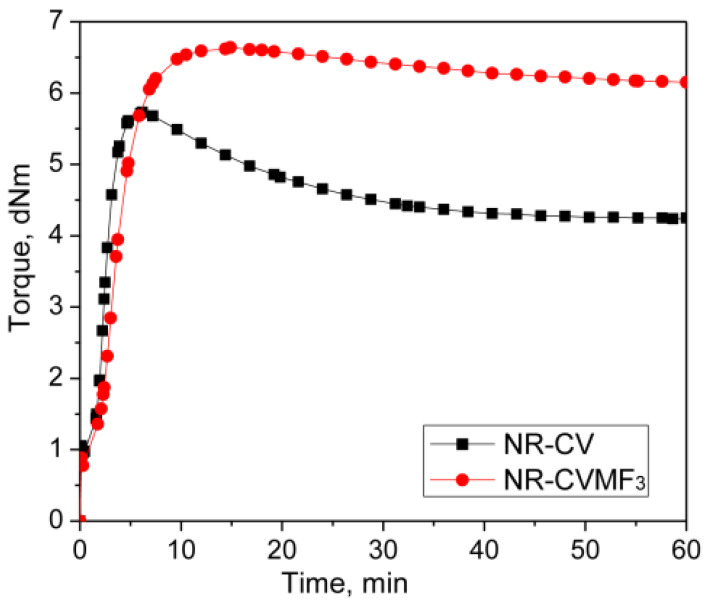
Curing curves of mixes 1 and 2 at 170 °C for 1 h.

Represented in Figure 2 are the curing curves of NR-CV and NR-CVMF_3_ at 170 °C for 1 h. Their cure characteristics are displayed in Table 2. NR-CV attains a maximum torque in 6.13 min and then exhibits a sharp declination in the rheometric torque with time due to reversion. The reversion was calculated and reported in percentage using Equation (4) and the values are depicted in Table 3.

(4) $$\mathrm{Reversion}\;(\%)=\frac{{S^{\prime }}_{max}-{S^{\prime }}_{60}}{{S^{\prime }}_{max}}\times 100$$

where *S′_max_* is the maximum torque and *S′*_60_ is the torque at 60 min.

The nature of the cure curve of NR-CVMF_3_ was almost similar to NR-CV during the initial stage of curing. However, NR-CVMF_3_ exhibits around 16% higher curing torque from the point where the reversion started in NR-CV. As a result, the state of cure in terms of Δ torque in NR-CVMF_3_ was improved by 19% compared to NR-CV.

From Table 3, it is clear that NR-CV exhibits around 26% reversion at the end of the given curing time. The intensity of reversion in NR-CV could be significantly reduced to 7.5% after incorporating 3 phr MF (NR-CVMF_3_). It has been reported that the curing process of diene rubbers with the CV system generates polysulfidic crosslinks in the cured network. These polysulfic crosslinks are unstable at elevated temperatures and rearrange to produce a certain amount of conjugated dienes on the rubber backbone, particularly at the point of reversion [14]. Therefore, the possibility of Diels–Alder reaction between the in situ formed conjugated diene at the point of reversion and the maleimide moieties of MF is responsible for the enhanced Δ torque during the curing of NR-CVMF_3_ [20,21,22]. The chemical stability of the bismaleimide-based bonds generated in the vulcanized network of NR-CVMF_3_ can be considered as its enhanced reversion resistance at elevated temperatures.

### 3.2. Curing Behaviors of Halogenated Elastomers with Metal Oxide and MF

**Figure 3:**
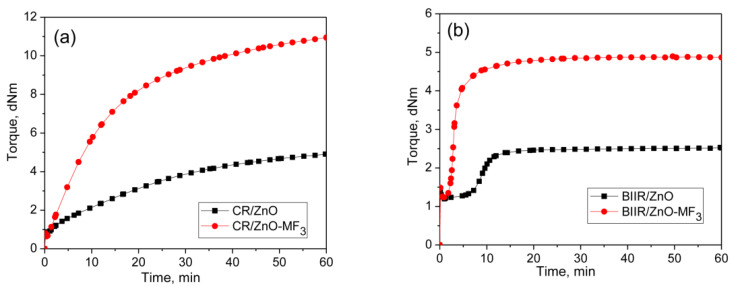
Curing curves of (**a**) Mixes 3 and 4 (**b**) mixes 5 and 6 at 170 °C for 1 h.

It is well-known that halogenated elastomers such as CR and BIIR can be cured with metal oxides. Represented in Figure 3a,b are the curing behaviors of CR and BIIR as per the formulations given in the mixes 3–6. Their cure characteristics are displayed in Table 2.

CR with metal oxide (ZnO/MgO) exhibit a marching modulus curing behavior with a high induction period (*T*_*S*2_ = 6.26 min) and time to optimum cure (*T*_90_ = 43.8 min). Similar behavior was also observed during the curing of BIIR with ZnO. Here also the induction period was very high (*T*_*S*2_ = 10.6 min). However, the curing of BIIR with ZnO exhibits a plateau type curing behavior. As a result, the *T*_90_ (13.9 min) of BIIR/ZnO was relatively low compared to CR/ZnO/MgO. It is interesting to note that the curing efficiency of ZnO in both the CR and BIIR becomes enhanced after incorporating 3 phr of MF. For instance, the extent of curing in terms of Δ torque in CR-ZnO has been improved by about 144% with the addition of 3 phr MF (CR-ZnO/MF_3_). Similarly, the Δ torque in BIIR/ZnO was improved by around 174% in the presence of 3 phr MF. Moreover, the induction period, *T*_*S*2_ of CR-ZnO has economically been reduced from 6.3 min to 2.2 min, and for BIIR/ZnO, it is reduced from 10.6 min to 2.7 min, respectively, after adding 3 phr MF. Literature reported that the halogenated rubbers such as CR and BIIR can also produce conjugated dienes when it is heating with ZnO [23,24]. Hence, the efficiency in the curing reaction between CR and BIIR with ZnO in the presence of MF can also be ascribed to the Diels–Alder reaction between the in situ formed dienes generated on the polymer with the maleimide moieties of MF [25,26,27].

### 3.3. Curing Behaviors of NR/CR (BIIR) Blends with CV and MF

From the curing behaviors of the virgin NR with CV/MF_3_ and the halogenated elastomers (CR and BIIR) with ZnO/MF_3_, it has been confirmed that MF can substantially improve the extent of curing by utilizing the in situ formed dienes because of the so-called Diels–Alder reaction. Therefore, an attempt has been made to exploit the in situ formed dienes from these elastomers to enhance the cure compatibility and other physico-mechanical properties of their blends.

**Figure 4:**
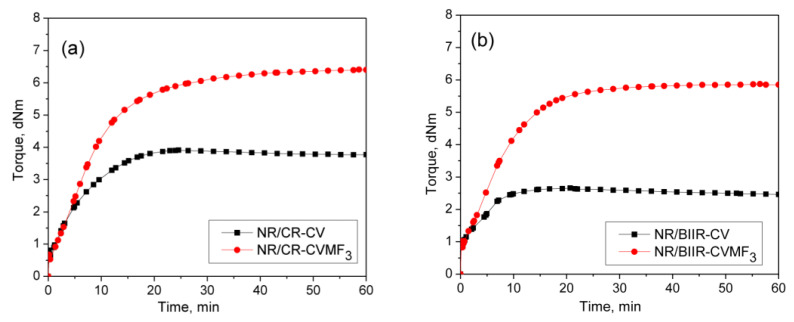
Curing curves of (**a**) mixes 7 and 8 (**b**) mixes 9 and 10 at 170 °C for 1 h.

**Figure 5:**
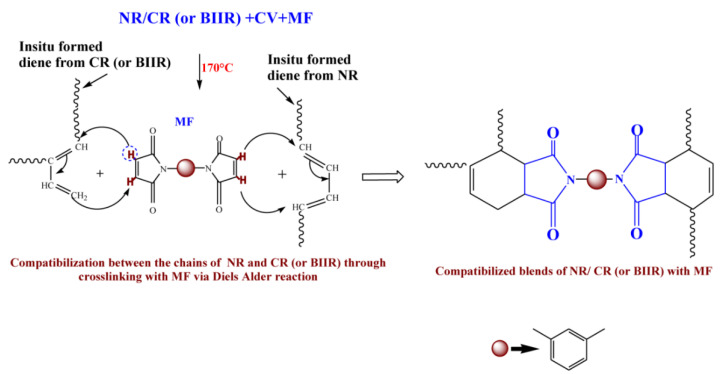
Plausible mechanism proposed for the compatibilisation (co-curing) effect of MF between NR and CR (BIIR) via Diels–Alder reaction.

To check whether MF can act as a compatibilizing (co-curing) agent, 50/50 blends of NR with CR and BIIR have been prepared as per the formulations corresponding to the mixes 7–10. Depicted in Figure 4a,b are the representative cure curves of NR/CR-CV, NR/CR-CVMF_3_, NR/BIIR-CV and NR/BIIR-CVMF_3_ at 170 °C for 1 h. Their cure characteristics are also displayed in Table 2. Both NR/CR-CV and NR/CR-CVMF_3_ exhibit almost the same speed of curing up to 4.4 min. Later, NR/CR-CV achieved a maximum torque of 3.91 dNm at about 25 min followed by a slight declination in the rheometric torque due to reversion. However, NR/CR-CVMF_3_ exhibits a marching modulus curing behavior and attained a maximum torque of 6.41 dNm nearly at the end of the given curing time (at 60 min). As a result, the extent of cure in terms of Δ torque in NR/CR-CVMF_3_ was 80% higher compared to NR/CR-CV. Because of the marching modulus curing behavior, the *T*_90_ of NR/CR-CVMF_3_ was higher than NR/CR-CV. Interestingly, no reversion was observed in NR/CR-CVMF3 till the end of the given curing time. The shape of the cure curves NR/BIIR-CV and NR/BIIR-CVMF_3_ depicted in Figure 4b were similar to NR/CR-CV and NR/CR-CVMF_3_, respectively. However, the speed of cure in terms of *T*_*S*2_ and the extent of cure in terms of Δ*M* in NR/BIIR-CV were much lower than NR/CR-CV. For instance, the *T*_*S*2_ and Δ*M* values of NR/CR-CV were 3.08 min and 3.27 dnM. On the other hand, the *T*_*S*2_ value of NR/BIIR-CV was 4.97 min and its Δ*M* was around 45% lower compared to NR/CR-CV. Here also, the NR/BIIR-CVMF_3_ exhibit a higher speed in the early stage of curing followed by a slight marching modulus curing behavior with a 182% higher extent of cure in terms of Δ*M* compared to NR/BIIR-CV. As in NR/CR-CVMF_3_, no reversion was also observed in NR/BIIR-CVMF_3_ till the end of the given 60 min of curing. The possibility of Diels–Alder reaction can be considered high to explain the enhanced state of cure in NR/CR-CVMF_3_ and NR/BIIR-CVMF_3_. From the knowledge of the curing behaviors of NR/CVMF_3_ and CR (BIIR)/ZnOMF_3_ as discussed earlier, it is reasonable to believe that the Diels Alder reaction might take place in NR/CR-CVMF_3_ or NR/BIIR-CVMF_3_ in three ways. One may be the in situ formed diene from the NR phase with the maleimide moieties of MF. The second one may be the in situ formed diene from the CR (or BIIR) phase with the maleimide moieties of MF. The third possibility could be a simultaneous Diels–Alder reaction between the dienes generated in the NR phase and CR (or BIIR) phase with either end of the maleimide moieties of MF as depicted in Figure 5. In this reaction, MF acts as a coupling/compatibilizing agent between NR and CR (or BIIR), which is essential to enhance the compatibility between these rubbers in their blends.

### 3.4. Cure-Strain Sweep Analysis of NR/CR (BIIR) with CV and MF 

**Figure 6:**
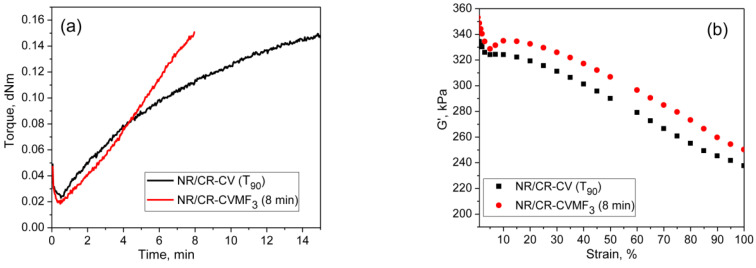

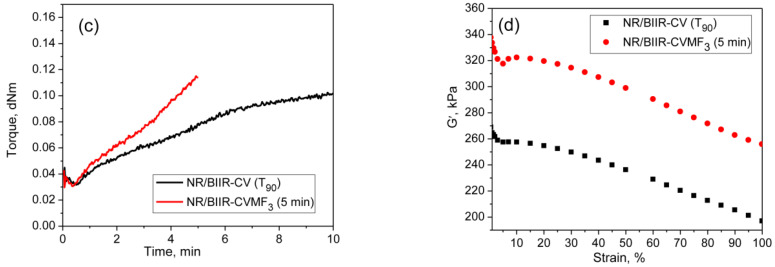
RPA cure curves at 170 °C and the corresponding strain-sweep curves at 40 °C of (**a**,**b**) *T*_90_ cured NR/CR-CV and 8 min cured NR/CR-CVMF_3_ and (**c**,**d**) *T*_90_ cured NR/BIIR-CV and 5 min cured NR/BIIR-CVMF_3_.

From the curing curves of the blends depicted in Figure 4a,b, it can be noticed that the torque corresponds to the *T*_90_ (15.2 min) of NR/CR-CV is 3.56 dNm. To reach the same level of torque, NR/CR-CVMF_3_ took only 8 min. Similarly, the torque at *T*_90_ (9.95 min) of NR/BIIR-CV is 2.48 dNm. To reach the same torque, NR/BIIR-CVMF_3_ took only 5 min. Based on the nature of these rheometer cure curves, it has been assumed that the strength of the cured network of the blends NR/CR-CVMF_3_ and NR/BIIR-CVMF_3_ might be higher than NR/CR-CV and NR/BIIR-CV, respectively, even if the magnitudes of their curing torque are the same. To check the validity of this assumption, a qualitative rheological test in the form of a cure-strain sweep was conducted as per the testing protocol described in the experimental Section 2.2.2 using the RPA. Depicted in Figure 6a are the RPA cure curves of NR/CR-CV up to its *T*_90_ (15 min) and NR/CR-CVMF_3_ up to 8 min at 170 °C. Their shear storage modulus (G′) vs. strain sweep curves are depicted in Figure 6b. It is well known that the rheological parameter G′ indicates the elastic response of viscoelastic material to an applied oscillatory strain. Hence, the term G′ can be considered as the strength of the cured network. Generally, the higher the G′ higher will be the strength of the cured network. It is interesting to note that at a given strain, the G′ of NR/CR-CVMF_3_ was considerably higher than NR/CR-CV even if the 15 min cured network of NR/CR-CV and the 8 min cured network of NR/CR-CVMF_3_ exhibited the same RPA Δ*M* of around 0.13 dNm. For instance, at 10% strain, the NR/CR-CV exhibits a G′ of 324 kPa. At the same strain, the G′ of NR/CR-CVMF_3_ was 3.7% higher than NR/CR-CV. Represented in Figure 6c,d are the RPA cure curves of NR/BIIR-CV up to its *T*_90_ (10 min) and NR/BIIR-CVMF_3_ cured up to 5 min at 170 °C and their G′ vs. strain sweep curves. As seen in the case of NR/CR-CV and NR/CR-CVMF_3_, similar behaviors were also observed in the G′ vs. strain sweep curves of NR/BIIR-CV and NR/BIIR-CVMF_3_. For instance, at 10% strain, the G′ of NR/BIIR-CVMF_3_ was around 25% higher than NR/BIIR-CV even though both the *T*_90_ cured NR/BIIR-CV and the 5 min cured NR/BIIR-CVMF_3_ exhibited the same RPA Δ*M* of around 0.07 dNm. This RPA cure-strain sweep results support the fact that MF can act as a compatibilising (co-curing) agent through the formation of bismaleimide adduct by utilizing the in situ generated dienes on the chains of NR and CIIR (or BIIR) via Diels–Alder reaction as described in Figure 5.

### 3.5. Swelling Behavior

**Figure 7:**
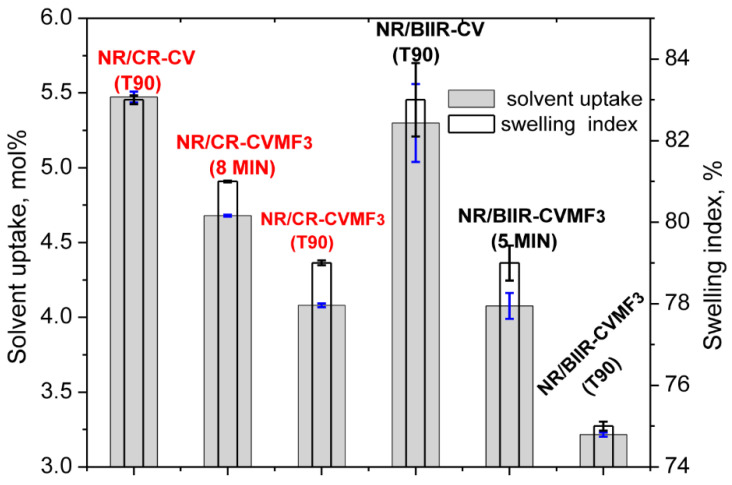
Equilibrium swelling index and the solvent uptake of the blends molded at different curing time.

Depicted in Figure 7 are the solvent uptake in mol% and the swelling index (percentage swelling) of the blends cured at different spans of time at 170 °C. 

It can be seen that the solvent uptake and the swelling index of the blend NR/CR-CV cured at its *T*_90_ was considerably higher than the corresponding NR/CR-CVMF_3_ cured at its *T*_90_. This swelling behavior is quite expected because the Δ*M* value of the *T*_90_ cured NR/CR-CV was around 44% lower than the *T*_90_ cured NR/CR-CVMF_3_. In general, the lower the Δ*M* value, the lower will be the crosslink density and, hence, the higher will be the solvent uptake and swelling index. However, it is interesting to note that the solvent uptake and the swelling index of NR/CR-CVMF_3_ cured up to 8 min was also lower than the *T*_90_ cured NR/CR-CV even though the Δ*M* produced in NR/CR-CVMF_3_ after 8 min of curing and the Δ*M* of NR/CR-CV cured up to its *T*_90_ exhibit almost the same value of around 3.0 dNm (Figure 2). Similarly, the solvent uptake of the *T*_90_ (10 min) cured NR/BIIR-CV was around 23 mol% higher than the 5 min cured NR/BIIR-CVMF_3_ even if both these blends cured up to the specified time exhibited the same Δ*M* of around 1.6 dNm.

**Figure 8:**
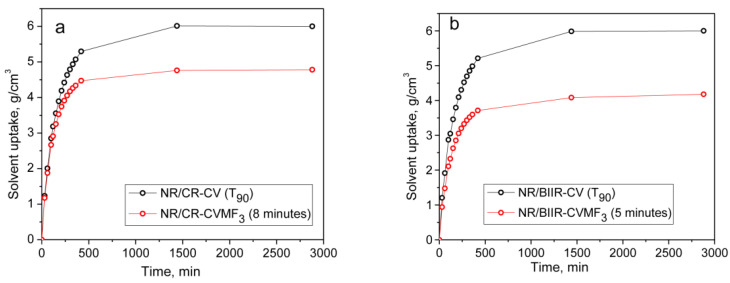
The solvent uptake of (**a**) *T*_90_ cured NR/CR-CV and 8 min cured NR/CR-CVMF_3_ (**b**) *T*_90_ cured NR/BIIR-CV and 5 min cured NR/BIIR-CVMF_3_.

From the above-mentioned equilibrium swelling studies of the blends, it has been confirmed that the blends cured with CVMF_3_ exhibit a higher swelling resistance in terms of solvent uptake compared to the blends cured with only CV. As already explained, one of the reasons for this might be a tightened network structure formed due to the formation of bismaleimide-based adducts between the chains of NR and CR (or BIIR). To check whether the network is really tightened or not right from the beginning of curing owing to the formation of the bismaleimide bonds as shown in Figure 5, we have monitored the solvent uptake of the blends at different intervals of time. Represented in Figure 8a,b is the solvent uptake in g/cm^3^ of the *T*_90_ cured NR/CR-CV, 8 min cured NR/CR-CVMF_3_, *T*_90_ cured NR/BIIR-CV and the 5 min cured NR/BIIR-CVMF_3_. It can be seen that the blend NR/CR-CV exhibits a higher speed of swelling in terms of solvent uptake compared to the NR/CR-CVMF_3_ even though both these blends cured up to the above-mentioned curing time exhibited the same magnitude of Δ*M*. For instance, after 5 min of swelling, the solvent uptake of the *T*_90_ cured NR/CR-CV was around 5% higher than NR/CR-CVMF_3_. Similarly, after 5 min of swelling, the solvent uptake of the *T*_90_ cured NR/BIIR-CV was around 25% higher than the 5 min cured NR/BIIR-CVMF_3_. This low amount of solvent uptake at the early stage of swelling of the blends cured with CVMF_3_ supports the fact that MF can act as a compatibilizer (co-curing agent) between NR and CR (or BIIR) by utilizing the in situ formed dienes via Diels–Alder reaction, as depicted in Figure 5.

### 3.6. Mechanical Properties

**Figure 9:**
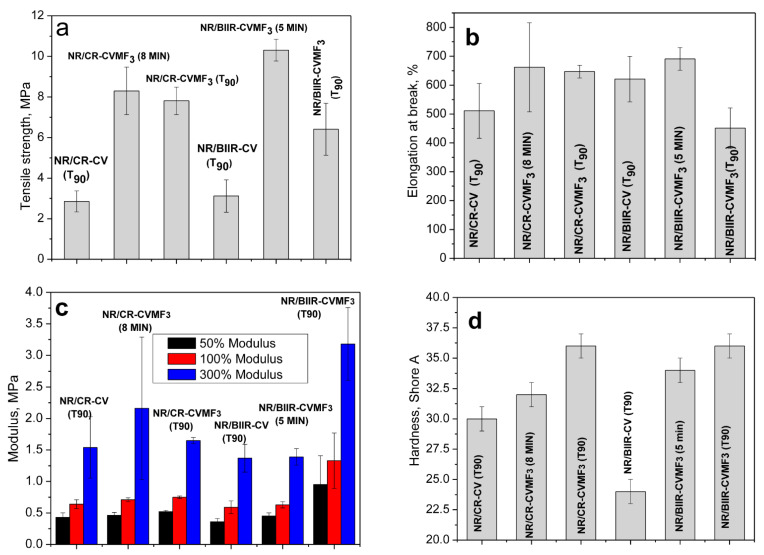
Mechanical properties of the blends moulded at different curing times (**a**) Tensile strength (**b**) Elongation at break (**c**) Modulus at different percentage elongations and (**d**) Shore-A hardness.

Depicted in Figure 9a–d are the mechanical properties such as the tensile strength (TS), elongation at break (EB), modulus at different percentage elongations and the shore-A hardness of NR/CR-CV, NR/CR-CVMF_3_, NR/BIIR-CV and NR/BIIR-CVMF_3_ molded at 170 °C as per their *T*_90_. NR/CR-CV shows a tensile strength of 2.85 MPa with a breaking elongation of 511%. However, the TS of NR/CR-CVMF_3_ was 174% higher than NR/CR-CV. Moreover, both the EB and modulus at different percentage elongations of NR/CR-CVMF_3_ were also considerably higher than NR/CR-CV. Similarly, the TS of NR/BIIR-CVMF_3_ was 107% higher than the corresponding NR/BIIR-CV. The modulus of NR/BIIR-CVMF_3_ at different percentage elongations were also significantly higher than NR/BIIR-CV. The hardness of the blends cured with CV and CVMF_3_ is represented in Figure 9d. The knowledge of the hardness of a rubber material is very essential, particularly when it is used in seals and gaskets. For sealing applications, the rubber compounds should be soft enough for better sealing ability, yet hard enough to sustain the loading force. Generally, rubbers with a tightly cross-linked network structure exhibit a high resistance to indentation. From Figure 9d, it is clear that the hardness of both the NR/CR-CVMF_3_ and NR/BIIR-CV MF_3_ were significantly improved compared to their respective NR/CR-CV and NR/BIIR-CV.

The above-mentioned mechanical properties were also evaluated after molding NR/CR-CVMF_3_ up to 8 min and NR/BIIR-CVMF3 up to 5 min. It is important to note that the Δ*M* value of the *T*_90_ cured NR/CR-CV and the 8 min cured NR/CR-CVMF_3_ are the same. Similarly, the Δ*M* value of the *T*_90_ cured NR/BIIR-CV and the 5 min cured NR/BIIR-CVMF_3_ are also the same. Interestingly, the TS of NR/CR-CVMF_3_ cured up to 8 min was around 191% higher than the *T*_90_ cured NR/CR-CV. Similarly, the TS of 5 min cured NR/BIIR-CVMF_3_ was 230% higher than the *T*_90_ cured NR/BIIR-CV. Moreover, the EB, modulus at different percentage elongations and shore-A hardness of the 8 min cured NR/CR-CVMF_3_ and the 5 min cured NR/BIIR-CVMF_3_ were considerably higher than the *T*_90_ cured NR/CR-CV and NR/BIIR-CV, respectively. These mechanical property data gives additional support for the enhanced compatibilization between NR and CR (or BIIR) with CVMF via Diels–Alder reaction.

## 4. Conclusions

The cure characteristics of 50/50 blends of NR/CR and NR/BIIR with a combination of conventional accelerated-sulphur (CV) and 3 phr of a bismaleimide (MF3) gives a strong indication that a co-curing has been taken place between NR/CR and NR/BIIR via Diels–Alder reaction. One of the primary pieces of evidence for this was the significant enhancement in the rheometer torque during the curing of these blends with CVMF_3_ compared to the same cured with only the CV system. Through Diles–Alder reaction, the rubber chains in their blends are interconnected via maleimide-based adducts. These crosslinks are believed to be stronger both mechanically and thermally than the sulphur crosslinks in the CV cured blends. Therefore, the networks formed by the CVMF_3_ cured blends were expected to be stronger than those cured with only the CV system. To check this further, the shear storage modulus (G′) of the blends were evaluated by conducting a specially designed cure-strain sweep analysis using a rubber process analyser. The results reveal that the G′ values of CVMF_3_ cured blends were higher than those cured with only the CV system, even though both the CVMF_3_ and the CV cured blends exhibited the same magnitude of crosslinking torque. This confirms the fact that the network formed in the CVMF_3_ cured blends is stronger than the CV cured blends. The reversion that was observed in the CV cured blends completely disappeared after curing these blends with CVMF_3_. This supports that the thermal stability of the CVMF_3_ cured blends was enhanced. The mechanical properties, particularly the tensile strength, modulus and hardness of the blends cured with CVMF_3_ exhibited significant improvements compared to the blends cured with only the CV system. Moreover, the swelling resistance of the CVMF_3_ cured blends improved significantly. All these results support the fact that the compatibility between NR and CR (BIIR) has enhanced in their blends via the proposed Diels–Alder reaction after curing them with CVMF_3_.

## Figures and Tables

**Table 1:** Formulation of the mixes.

Mix No.	Mix ID	NR	CR	BIIR	ZnO	MgO	St. Acid	Sulfur	CBS	MF
1	NR-CV	100	-	-	5	-	2	2.5	0.5	-
2	NR-CVMF_3_	100	-	-	5	-	2	2.5	0.5	3
3	CR-ZnO	-	100	-	5	4	0.5	-	-	-
4	CR-ZnOMF_3_	-	100	-	5	4	0.5	-	-	3
5	BIIR-ZnO	-	-	100	5	-	-	-	-	-
6	BIIR-ZnOMF_3_		-	100	5	-	-	-	-	3
7	NR/CR-CV	50	50	-	5	2	1.25	1.25	0.25	-
8	NR/CR-CVMF_3_	50	50	-	5	2	1.25	1.25	0.25	3
9	NR/BIIR-CV	50	-	50	5	-	1	1.25	0.25	-
10	NR/BIIR-CVMF_3_	50	-	50	5	-	1	1.25	0.25	3

**Table 2:** Cure characteristics of the mixes at 170 °C, 1 h.

Mix ID	M_L_ (dNm)	M_H_ (dNm)	Δ*M* (dNm)	*T*_*S*2_ (min)	*T*_90_ (min)	CRI (min^−1^)
NR-CV	0.96	5.73	4.77	1.95	3.88	51.81
NR-CV-MF_3_	0.77	6.64	5.87	2.32	6.86	22.02
CR-ZnO	0.74	4.90	4.16	6.26	43.83	2.66
CR-ZnO-MF_3_	0.64	10.14	9.50	2.18	37.25	2.85
BIIR-ZnO	1.20	2.53	1.33	10.64	13.92	30.48
BIIR-ZnO-MF_3_	1.24	4.89	3.65	2.71	8.89	16.18
NR/CR-CV	0.64	3.91	3.27	3.08	15.18	8.26
NR/CR-CV-MF_3_	0.53	6.41	5.88	2.92	22.36	5.14
NR/BIIR-CV	0.87	2.66	1.79	4.97	9.95	20.08
NR/BIIR-CV-MF_3_	0.82	5.87	5.05	3.10	18.05	6.68

**Table 3:** Reversion in compounds at 170 °C.

Mix ID	Reversion (%)
NR-CV	25.8
NR-CVMF_3_	7.5
NR/CR-CV	3.83
NR/CR-CVMF_3_	No reversion
NR/BIIR-CV	7.9
NR/BIIR-CVMF_3_	No reversion

## Data Availability

The data presented in this study are available on request from the corresponding author.
